# Impact of Sex and Excess Weight on the Risk of Colorectal Cancer

**DOI:** 10.1007/s10620-025-09526-6

**Published:** 2025-11-04

**Authors:** Hermann Brenner, Luna Kiran Adhikari, Fatemeh Safizadeh

**Affiliations:** 1 German Cancer Research Center (DKFZ), Im Neuenheimer Feld 280, 69120 Heidelberg, Germany; 2 German Cancer Research Center (DKFZ), Heidelberg, Germany; 3 Institute for Medical Information Processing, Biometry and Epidemiology – IBE, LMU Munich, Munich, Germany; 4 Pettenkofer School of Public Health, Munich, Germany

The disquieting rise of the incidence of early onset colorectal cancer (EOCRC) observed in many countries [1] has prompted major research activities aimed to better understand its risk factors and how they might differ from those for late-onset colorectal cancer (LOCRC). In their analyses of the UK Biobank data, Sandhu et al. aimed to quantify the impact of risk factors of EOCRC and LOCRC [2]. They did so by calculating adjusted odds ratios (ORs) for defined candidate risk factors in matched case–control designs, using nearest neighbor matching based on Euclidean distance for age and exact matching for sex. Among other results, apparent main findings (also highlighted in the abstract) were that male sex seemed to be associated with a reduced risk of LOCRC (OR 0.78, 95% CI 0.71–0.86), whereas waist-to-hip ratio (WHR) seemed to be associated with an exceptionally strong risk for LOCRC (OR 5.81; 95% CI 3.25–10.38) in multivariable analyses. Both results deserve critical comment and interpretation.

The apparent protective effect of male sex against LOCRC, which is in sharp contrast to well-established evidence from a wealth of epidemiological studies and the almost universally seen higher LOCRC incidence rates of men compared to women [3], is strongly misleading. Matching for sex, as reported by Sandhu et al., implies that ORs for sex cannot be interpreted as reflecting sex effects any longer. In fact, matching seems to have worked as intended in the study by Sandhu et al., as reflected in the identical age and sex distributions of cases and controls shown in their Table 1 which would result in crude ORs of 1.00 in crude analysies.

For proper interpretation of the OR for the association of WHR with LOCRC, information would be needed for which comparison of WHR categories, or—in case of using WHR as continuous variable—for which increase in WHR the OR is reported. Associations for continuous variables should be reported for meaningful, easy-to-interpret and easy-to-compare increases, such as an increase by one standard deviation. Sandhu et al. do not report which difference in WHR their reported OR refers to. The magnitude of the association suggests that WHR was included as a continuous variable, and that the reported OR reflects the risk with a one unit increase in WHR. However, the standard deviation of WHR equals approximately 0.1 only, as observed in previous analyses of the UK Biobank data [4]. This would imply that the exceptionally high OR reported by Sandhu et al. refers to an extreme contrast of WHR by approximately 10 standard deviations. Recalculating an expected OR for a one standard deviation increase would yield an OR of exp(ln(5.81)/10) = 1.19, with a 95% confidence interval of (exp(ln(3.25)/10), exp(ln(10.38)/10)) = (1.13,1.26). These values are very close to effect estimates previously reported for the risk of CRC associated with a one standard deviation increase in WHR using the UK Biobank data [4]. Concerns regarding reported ORs per one unit increase also apply to ORs for other variables, such as telomere length.

In addition, besides being of an uninterpretable magnitude, the association of WHR with EOCRC shown in Table 2 by Sandhu et al. has a curiously large confidence interval (CI) from 0.02 to 379.03. This serious instability in estimation most likely reflects a major collinearity problem due to the high correlation between BMI and WHR.

Finally, the statement by Sandhu et al. that “data on hypertension, hyperlipidemia, and diabetes were only available for males, not females” suggests that there may have been problems with the dataset used by Sandhu et al., which may have distorted the analyses. Many papers have been published on these factors showing their distributions in males and females in the UK Biobank [e.g., 5].

In summary, the effect estimates reported by Sandhu et al. need to be interpreted with due caution. Besides other methodological concerns, the matched design along with inappropriate analysis led to invalid results for sex, while results for WHR seem to be strongly exaggerated due to comparison of outlier values approximately 10 standard deviations apart.

## Data Availability

No datasets were generated or analysed during the current study.

## References

[1] Siegel RL, Wagle NS, Cercek A, Smith RA, Jemal A. Colorectal cancer statistics, 2023. *CA Cancer J Clin.* 2023;73:233–254. 36856579 10.3322/caac.21772

[2] Sandhu S, Blandon C, Kumar S. Evaluating risk factors for early-onset colorectal cancer in a large, prospective cohort. *Dig Dis Sci.* 2025;70:2834–2842. 10.1007/s10620-025-09055-2. 40234296 10.1007/s10620-025-09055-2 PMC12367846

[3] Brenner H, Hoffmeister M, Arndt V, Haug U. Gender differences in colorectal cancer: implications for age at initiation of screening. *Br J Cancer.* 2007;96:828–831. 17311019 10.1038/sj.bjc.6603628 PMC2360074

[4] Safizadeh F, Mandic M, Schöttker B, Hoffmeister M, Brenner H. Central obesity may account for most of the colorectal cancer risk linked to obesity: evidence from the UK Biobank prospective cohort. *Int J Obes (Lond).* 2025;49:619–626. 39562688 10.1038/s41366-024-01680-7 PMC11999858

[5] Fry A, Littlejohns TJ, Sudlow C, Doherty N, Adamska L, Sprosen T et al. Comparison of sociodemographic and health-related characteristics of UK Biobank participants with those of the general population. *Am J Epidemiol.* 2017;186:1026–1034. 28641372 10.1093/aje/kwx246 PMC5860371

